# Health-related quality of life in post-stroke patients attended at tertiary-level hospitals in Bangladesh

**DOI:** 10.3389/fstro.2024.1411422

**Published:** 2024-06-27

**Authors:** Mohammad Jahirul Islam, Sohel Ahmed, Samena Akter Kakuli, Mohammad Habibur Rahman, Sharker Md. Numan, Shishir Ranjan Chakraborty, Md. Jamal Uddin, Manzur Kader

**Affiliations:** ^1^Department of Physical Medicine and Rehabilitation, MAG Osmani Medical College Hospital, Sylhet, Bangladesh; ^2^Ahmed Physiotherapy and Research Center, Dhaka, Bangladesh; ^3^Directorate of Students' Welfare, Bangladesh University of Engineering and Technology, Dhaka, Bangladesh; ^4^School of Science and Technology, Bangladesh Open University, Gazipur, Bangladesh; ^5^Department of Medicine, MAG Osmani Medical College Hospital, Sylhet, Bangladesh; ^6^Department of Statistics, Shahjalal University of Science and Technology, Sylhet, Bangladesh; ^7^Faculty of Graduate Studies, Daffodil International University, Dhaka, Bangladesh; ^8^Department of Medical Science, School of Health and Welfare, Dalarna University, Falun, Sweden

**Keywords:** stroke, quality of life, cross-sectional study, rehabilitation, Bangladesh

## Abstract

**Background:**

Insufficient data on the health-related quality of life (HRQoL) of stroke survivors in less-resourced regions like Bangladesh emphasizes the need for understanding influencing factors. In this cross-sectional study, our objective was to assess potential factors associated with the HRQoL among stroke survivors in Bangladesh.

**Methods:**

The study included 424 stroke survivors (65% male, mean age 57.25 ± 12.13 years) undergoing rehabilitation at four tertiary-level hospitals in Bangladesh. The HRQoL was assessed using the European Quality of Life Scale-5 Dimensions (EuroQol-5D), covering mobility, self-care, usual activities, pain/discomfort, and anxiety/depression, along with a visual analog scale (VAS). Sociodemographic such as age, marital status, education, occupation, tobacco habit cohabitant situation, and health-related factors such as type and duration of stroke, co-morbidity, receipt of rehabilitation, and use of assistive devices served as independent variables. Bivariate logistic regression was utilized to ascertain the estimated risk factors for HRQOL, presenting odds ratios (OR) and a 95% confidence interval (CI) after adjusting for potential confounders.

**Results:**

The study primarily involved participants from rural areas (57.8%) with primary education (67.7%). Stroke survivors reported a mean EQ summary index of 0.393 ± 0.46 and a VAS score of 40.43 ± 18. A majority experienced a stroke within 1–3 months (66%), with 52.6% exhibiting left-side weakness. The results highlight significant challenges among survivors: 79.5% faced mobility issues, 81.1% were dependent in self-care, 87% had activity limitations, 70.8% suffered from bodily pain, and 84% experienced symptoms of anxiety or depression. Widowed or single survivors encountered greater difficulties in mobility (Adjusted Odds Ratio, AOR = 1.24, 95% CI = 0.35–4.45) and pain/discomfort (AOR = 2.85, 95% CI = 0.85–9.27) compared to their married counterparts. Those lacking access to rehabilitation services faced considerably higher challenges: mobility difficulties were nearly thirty times greater (AOR = 29.37, 95% CI = 8.85–97.50), difficulties in self-care were about forty-four times higher (AOR = 43.21, 95% CI = 10.02–186.41), challenges in usual activities were also forty-four times more frequent (AOR = 43.47, 95% CI = 5.88–321.65), pain/discomfort was nearly five times more prevalent (AOR = 4.35, 95% CI = 2.45–7.71), and anxiety or depression was over twenty times more common (AOR = 20.14, 95% CI = 7.21–56.35) compared to those who received rehabilitation services.

**Conclusion:**

The findings suggest that the enhancement of HRQoL in post-stroke patients in Bangladesh necessitates targeted interventions, including family support, tobacco cessation, recurrent stroke prevention, and effective rehabilitation services. Longitudinal studies are recommended for further confirmation of these findings.

## 1 Introduction

Stroke is emerging as one of the leading causes of disability on a global scale (Johnson et al., 2019). The Global Burden of Disease (GBD) study in 2019 reported 12.2 million incident cases of stroke, resulting in the loss of 143 million disability-adjusted life years (DALYs), impacting both developed and developing nations (Feigin et al., 2021). In South Asia, stroke led to ~1 million deaths and 22 million lost DALYs, with Bangladesh alone experiencing 2.8 million lost DALYs (Johnson et al., 2019). A population-based study indicated a stroke prevalence of 2.52% in adults aged 50–60 and 3.01% in those over 60 in Bangladesh (Alam et al., 2022). Stroke significantly affects survivors' physical, mental, and psychosocial dimensions, diminishing functional capacity and severely impacting Health-Related Quality of Life (HRQOL) (Kariyawasam et al., 2020). Therefore, planning for the restoration of physical function and independence after post-stroke is crucial (Langhorne et al., 2011) Additionally, enhancing HRQOL is integral to stroke treatment and rehabilitation (Carod-Artal and Egido, 2009), and identifying factors associated with HRQOL is crucial for developing an effective treatment and rehabilitation strategy.

The World Health Organization defines quality of life (QoL) as an individual's perception of their position in life within cultural and value systems, considering their goals (WHO, 2012). Various validated tools exist for QoL assessment, including the European Quality of Life Scale-5 Dimensions (EQ-5D-5L), which evaluates mobility, self-care, usual activities, pain/discomfort, and anxiety/depression (Golicki et al., 2015). In EQ-5D assessments, stroke patients with lower income, younger age, unemployment, smoking habits, recurrent stroke, lower physical activity, co-morbidities, anxiety, depression, and poor sleep quality showed a significant association with lower HRQOL in studies conducted in China (Mei et al., 2022) and Malaysia (Wong et al., 2021) A study in Pakistan found that higher disability, lower functional level, and stroke severity were linked to lower HRQOL (Khalid et al., 2016). However, a recent scoping review highlights a gap in HRQOL research post-stroke, noting a concentration of studies in affluent countries, potentially overlooking populations in less-resourced regions like Bangladesh (Gurková et al., 2023). In Bangladesh, the challenge is compounded by the limited accessibility of public health facilities for individuals with disabilities, including stroke survivors (Torsha et al., 2022). The country has about 6.8 rehabilitation units per million people, with a mere 6.2% located in rural areas. The rehabilitation workforce includes 9.4 physiotherapists, 1.3 occupational therapists, and 0.9 speech and language therapists per million people. A significant barrier is the financial burden on patients, as 66.3% of rehabilitation services require out-of-pocket payments (Al Imam et al., 2022). These factors collectively hinder effective stroke rehabilitation and negatively impact HRQOL outcomes. Addressing this research deficiency, our study investigates HRQOL among stroke survivors in Bangladesh using the EQ-5D-5L, a tool that has not been previously applied in this context. This research aims to provide localized insights into the recovery outcomes of Bangladeshi stroke patients, spotlighting unique challenges such as accessibility to rehabilitation facilities and the affordability of services. By offering data that supports tailored rehabilitation strategies, this study aims to enhance global understanding and aid in policy formulation to improve life quality outcomes for this vulnerable group.

Ultimately, this study focuses on assessing the HRQOL of patients 1-month post-stroke, aiming to provide valuable insights that will help establish relevant rehabilitation goals and strategies tailored for the Bangladeshi context. The findings are expected to be instrumental in informing both clinical practices and health policy, thereby improving the support and care provided to stroke survivors in Bangladesh.

## 2 Methodology

### 2.1 Study design

This cross-sectional study employed a convenience sampling method and was carried out between January and June 2022. Prospective participants were recruited from individuals accessing rehabilitation services at the physiotherapy and rehabilitation department.

#### 2.1.1 Study population

Individual who had experienced a stroke at least 1 month prior to their inclusion in the study. The research followed the 2013 Helsinki Declaration protocols and obtained ethical clearance from the Bangladesh Open University Ethics Committee (BOU/SST/MDMR/146/17/376).

### 2.2 Sample and sample size estimation

The sampling procedure for the study was done using the following equation: Z^2^pq/d^2^. Where *Z*^2^ = 1.96, *p* = the expected health status by EQ-VAS is 60 % (Wong et al., 2021), *d* = 5% margin of error, and the attrition rate is 15% to avoid bias. Applying the formula yielded a minimum sample size of 424.

### 2.3 Eligibility criteria

#### 2.3.1 Inclusion criteria

Seeking stroke patients, aged 18–80, with confirmed ischemic or hemorrhagic stroke diagnosed via CT scan or MRI, occurring at least 1 month ago. Participants should have no preexisting stroke-related disability (per mRS), and willingness to engage in the study is essential.

#### 2.3.2 Exclusion criteria

Exclusion criteria encompassed global aphasia and/or communication inability, medical instability, concurrent neurological disorders (brain tumor, traumatic brain injury, mental disorder, Parkinson's disease, dementia), pre-existing musculoskeletal conditions (spinal cord injury, hip fracture, dislocations, contractures), ongoing psychoactive substance abuse, pre- and post-stroke psychiatric morbidity, and terminal illnesses like renal failure or end-stage cancer (Khalid et al., 2016).

### 2.4 Data collection procedures

An interviewer-administered questionnaire was employed to collect data, translated into Bangla and back into English with expert input. A pretest involved sixteen respondents outside the study area in a similar setting. The interview was conducted by four trained data collectors (physiotherapy graduates with >3 years of experience). Before beginning data collection, all interviewers completed a training session led by the principal researcher to ensure uniformity in their approach to different interviewees. Each data collection session was held in a consistent setting, where interviewers were instructed to collect five specific data points and promptly resolve any issues that arose. Additionally, clinical information, including type of stroke, side of weakness, duration of a stroke, and co-morbidity, was extracted from the patient's medical records. Data collectors, who received thorough training, conducted in-person interviews with participants. Initially, a data collector presented a question to the respondent, and upon receiving a response, the collector repeated the respondent's answer for confirmation to ensure accuracy and understanding. In order to minimize selection bias and ensure a diverse sample, this study enlisted participants from four distinct tertiary care hospitals, including one government medical college hospital, one private medical college hospital, and two private tertiary care hospital. This approach allowed for the inclusion of individuals from various geographical locations and socio-economic backgrounds.

#### 2.4.1 Data collection tools

The questionnaire encompasses socio-demographic inquiries pertaining to age, sex, residence, educational status, marital status, occupation, income, smoking, tobacco habits, cohabitant situation, and care provider. Clinical profile included the type of stroke, side of weakness, duration of a stroke, co-morbidity, the person responsible for medical expenses, receiving rehabilitation care, and assistive device use.

##### 2.4.1.1 EuroQL-5

This study utilized a modified and translated Bengali iteration of the EQ-5D-5L health-related quality of life questionnaire. The questionnaire used in our study was transculturally validated, and details about its availability and various versions can be found on the EuroQol website at https://euroqol.org/register/obtain-eq-5d/available-versions/. This version of the questionnaire has been applied in various Bangladeshi populations, including patients with diabetes mellitus (Saleh et al., 2015). The instrument comprises two integral components: the EQ-5D descriptive version and the EQ visual analog scale (EQ-VAS). The EQ-5D-5L description system encompasses five dimensions: mobility, self-care, usual activities, pain or discomfort, and anxiety or depression. Participants were instructed to evaluate their present health status using a 5-point Likert scale, wherein a score of 1 denoted the absence of problems, and a score of 5 signified significant problems. Respondents were also asked to assess “their health today” on a 0–100 vertical-thermometer-like scale (worst to best score) using EQ-VAS (Dolan, 1997; Shafie et al., 2019; Wong et al., 2021). In our study, we computed the EQ summary index by assigning numerical values (ranging from 1 to 5) to denote the five levels of health across each of the five dimensions measured by EQ-5D-5L. These health states were then transformed into a consolidated measure known as utility, represented on a scale from −1 to 1. Here, 0 signifies death, values <0 indicate states worse than death, and 1 represents full health. To derive the EQ index values, we employed the calculation method established by Jyani et al. (Jyani et al., 2022) in India, given the absence of a standard value set for Bangladesh and the geographical similarity between the two neighboring countries.

##### 2.4.1.2 The modified Rankin Scale

mRS is frequently employed to assess disability post-stroke, aligning with the International Classification of Functioning, Disability, and Health (ICF) model encompassing body function, activity, and participation. The scale is a 7-point system ranging from 0 (no symptoms, completely normal) to 5 (severe disability), with six denoting death. Notably, the scale exhibits robust test-retest reliability, with a range of *k* = 0.81 to 0.95 (Banks and Marotta, 2007).

### 2.5 Measurement of variables

#### 2.5.1 Independent variables

Initially, age was considered a continuous variable; however, it was subsequently categorized into three groups: 18–40 years, 41–60 years, and 61–80 years. Other variables were also categorized as follows: gender (male/female), place of residence (urban/rural), marital status (single or widowed/married), level of education (primary/secondary/tertiary), occupation (employed, including service, business, farming, daily labor; unemployed, including homemakers, retired, others), monthly family income (15,000 taka/15,000–30,000 taka/31,000–45,000 taka/>45,000 taka), smoking (No/Yes), tobacco habit (No/Yes), cohabitation situation (spouse & children/others, including parents, siblings, relatives), care providers (spouse & children/others), type of stroke (ischemic/hemorrhagic), duration of stroke (1–3 months/3–6 months/>6 months), side of weakness (right/left/both sides), number of stroke episodes (1st time/recurrent), co-morbidity (No/Yes), receiving rehabilitation care (No/Yes), the medical expenses provided by (self or spouse/children/others), and using assistive devices in daily life (No/Yes).

#### 2.5.2 Dependent variables

##### 2.5.2.1 The modified Rankin Scale

This scale is a 7-point scale categorized from 0 = normal, 1 = no significant disability, 2 = slight disability, 3 = moderate disability, 4 = moderately severe disability, 5 = severe disability and 6 = death.

##### 2.5.2.2 EuroQL-5

The EQ-5D descriptive version of the EQ-5D-5L (mobility, self-care, usual activities, pain/discomfort, and anxiety/depression) scores 1 = no problem, 2 = slight problems, 3 = moderate problems, 4 = severe problems, and 5 = extreme problems and the EQ visual analog scale (EQ-VAS). Participants were asked to evaluate their current health using a vertical-thermometer-like scale ranging from 0 to 100 (from worst to best score). Furthermore, we calculated the EQ-5D summary index, with values between-−0.923 and 1 (0 indicating death and 1 indicating full health). A score below 0 is considered a health status worse than death.

### 2.6 Data analysis

The data was analyzed using SPSS-25 software to perform descriptive and inferential statistical analyses. Continuous variables were characterized using means and standard deviations (SD), while categorical variables were represented using frequencies and percentages. Univariate analysis employed the chi-square test and Fisher exact test to explore potential associations among socio-demographic status, clinical profiles, and EQ-5D dimensions. Consistent with previous research, it was noted that the EQ-5D instrument employs a pair of categorical variables for each domain (Wong et al., 2021). These variables were characterized by two discrete states: (a) absence of issues and (b) presence of problems categorized into degrees of severity (slight, moderate, severe, and extreme). The EQ summary index was subjected to statistical analyses, employing the Independent Samples *T*-test for two groups and the one-way Analysis of Variance (ANOVA) test for more than two groups. Multiple binary Logistic regression was used to ascertain the estimated risk factor for Health-Related Quality of Life (HRQOL) among stroke survivors. This involved incorporating independent variables with a *p*-value <0.05 from the univariate analysis and using Adjusted Odds Ratios (AOR) and a 95% confidence interval (CI). The fitness of model for each EQ dimension was check by Hosmer-Lemshow's test and classification table. The variance inflation factors (VIF) was used to test for multicollinearity amongst the independents variables (VIF = <5.0) (O'brien, 2007). The researchers established a significance level of <0.05.

## 3 Results

### 3.1 Socio-demographic characteristics and clinical profile of respondents

This study involved 424 participants, with an average age of 57.25 ± 12.13 years. The majority were male (64.9%), married (80.9%), residing in rural areas (57.8%), and had completed only primary education (67.7%). Notably, 66% experienced a stroke within 1–3 months, and 52.6% reported left-side weakness. The participants displayed a high prevalence of various co-morbidities. For a comprehensive overview of the respondents' clinical and socio-demographic details, refer to Table 1.

**Table 1:** Socio-demographic characteristics of respondents (*n* = 424).

**Variable**		**Frequency**	**Percentage**
Age (in years)	18–40	48	11.3
	41–60	214	50.5
	61–80	162	38.2
Gender	Male	275	64.9
	Female	149	35.1
Residence	Rural	314	74.1
	Urban	110	25.9
Marital status	Single/widow	81	19.1
	Married	343	80.9
Education level	Primary	287	67.7
	Secondary	93	21.9
	Tertiary	44	10.4
Occupation	Service	52	12.3
	Business	76	17.9
	Farming	70	16.5
	Homemaker	114	26.9
	Retired/unemployed	112	26.4
Monthly family income (in taka)	<15,000	65	15.3
	15,000–30,000	200	47.2
	31,000–45,000	113	26.7
	>45,000	46	10.8
Smoking habit	No	228	53.8
	Yes	196	46.2
Tobacco habit	No	217	52.2
	Yes	207	48.8
Co-habitant situation	Spouse and children	321	75.7
	Others (parents/sibling/ relatives)	103	24.3
Care provided by	Spouse and children	233	55
	Others (parents/sibling/ relatives)	191	45
Type of stroke	Ischemic	357	84.2
	Hemorrhage	67	15.8
Duration of stroke	1–3 months	280	66
	4–6 months	58	13.7
	>6 months	86	20.3
Side of weakness	Right side	191	45
	Left side	223	52.6
	Both side	10	2.4
Number of stroke episodes	1st episode	335	79
	Recurrent	89	21
Co-morbidity	No	58	13.7
	Yes	366	86.3
Type of co-morbidity	Diabetes	166	39.2
	Hypertension	286	67.5
	Diabetes and hypertension	128	30.2
Undergo any rehabilitation service	No	158	37.3
	Yes	266	62.7
Medical expenses provided by	Self/spouse	139	32.8
	Children	254	59.9
	others	31	7.3
Use of an assistive device	No	316	74.5
	Yes	108	25.5

### 3.2 The severity of problems in each EQ-5D-5L dimension among stroke survivors

The HLQOL among stroke survivors is significantly impacted, as evidenced by key findings. A notable 79.5% of participants had mobility problems, 81.1% could not carry out personal self-care, 87% could not perform their usual activity, 70.8% had pain in their bodies, and 84% experienced anxiety or depression. Table 2 provides the information in detail.

**Table 2:** Severity of problems in each EQ-5D-5L dimension among stroke survivors (*n* = 424).

**Variables**	**No problems (%)**	**Slight problems (%)**	**Moderate problems (%)**	**Severe problems (%)**	**Extreme problems (%)**
Mobility	87 (20.5)	100 (23.6)	118 (27.8)	71 (16.7)	48 (11.3)
Self-care	80 (18.9)	71 (16.7)	94 (22.2)	97 (22.9)	82 (19.3)
Usual activities	55 (13)	85 (20)	76 (17.9)	118 (27.8)	90 (21.2)
Pain/discomfort	124 (29.2)	185 (43.6)	94 (22.2)	16 (3.8)	5 (1.2)
Anxiety/depression	68 (16)	98 (23.1)	157 (37)	90 (21.2)	11 (2.6)

### 3.3 Association between socio-demographic and clinical variables with EQ-5D-5L and EQ utility index

A significant association existed between the number of stroke episodes experienced, the use of rehabilitation care, and the utilization of an assistive device, along with all physical dimensions of the EQ-5D-5L. Relationships with the EQ mobility component were found to be significant for age, marital status, tobacco habit, care providers, and medical expense providers. The EQ self-care dimension showed significant relationships with variables such as marital status, monthly family income, tobacco habit, cohabitant situation, care providers, and medical expense providers. A robust correlation was noted between EQ-usual activities and factors such as residential neighborhood, length of stroke, and care providers. The EQ-pain/discomfort dimension showed significant associations with age, marital status, education level, occupation, tobacco habit, co-morbidity, type of stroke, duration of stroke, cohabitant situation, and medical expense providers. Additionally, significant associations were identified between respondents' education level, monthly family income, tobacco intake, and medical expense providers with the EQ-anxiety/depression dimension. Detailed information is provided in Table 3.

**Table 3:** Association between socio-demographics, and clinical profiles with EQ-5D, utility index and VAS score.

**Variables**	***n* (%)**	**Mobility problems**	**Self-care problems**	**Usual activities problems**	**Pain/ discomfort problems**	**Anxiety/ depression problems**	**Mean utility index (SD)**	**Mean VAS Score (SD)**
**Age (years)**								
18–40	48 (11.3)	41 (85.4)	43 (89.6)	43 (89.6)	28 (58.3)	45 (93.8)	0.40 (0.45)	39.81 (18.11)
41–60	214 (50.5)	159 (74.3)	166 (77.6)	183 (85.5)	148 (69.2)	175 (81.8)	0.48 (0.48)	43.07 (18.09)
61–80	162 (38.2)	136 (84)	136 (84)	143 (88.3)	124 (76.5)	136 (84)	0.27 (0.47)	37.12 (17.61)
***P*** **-value**		**0.039** ^*^	0.087	0.627	**0.039** ^*^	0.118	**<0.001** ^**^	**0.006** ^*^
**Gender**								
Male	275 (64.9)	211 (76.7)	218 (79.3)	237 (86.2)	189 (68.7)	229 (83.3)	0.43 (0.44)	40.34 (17.77)
Female	149 (35.1)	125 (83.9)	127 (85.2)	132 (88.6)	111 (74.5)	127 (85.2)	0.31 (0.49)	40.59 (18.71)
***P*** **-value**		0.082	0.132	0.481	0.212	0.599	**0.015** ^*^	0.891
**Residence**								
Urban	110 (25.9)	83 (75.5)	85 (77.3)	90 (81.8)	75 (68.2)	95 (86.4)	0.42 (0.53)	45.56 (20.62)
Rural	314 (74.1)	253 (80.6)	260 (82.8)	279 (88.9)	225 (71.7)	261 (83.1)	0.38 (.43)	38.63 (16.77)
***P*** **-value**		0.255	0.2	0.059	0.491	0.425	0.499	**0.002** ^*^
**Marital status**								
Single/widow	81 (19.1)	71 (87.7)	72 (88.9)	75 (92.6)	67 (82.7)	72 (88.9)	0.26 (0.44)	41.36 (18.88)
Married	343 (80.9)	265 (77.3)	265 (77.3)	294 (85.7)	233 (67.9)	284 (82.8)	0.42 (0.46)	36.46 (13.59)
***P*** **-value**		**0.038** ^*^	0.053	0.097	**0.009** ^*^	0.179	**0.005** ^*^	**0.008** ^*^
**Level of education**								
≤ Primary	287 (67.7)	235 (81.9)	239 (83.3)	252 (87.8)	212 (73.9)	246 (85.7)	0.34 (0.46)	37.84 (16.82)
Secondary	93 (21.9)	67 (72)	73 (78.5)	79 (84.9)	57 (61.3)	71 (76.3)	0.43 (0.49)	42.81 (18.96)
Tertiary	44 (10.4)	34 (77.3)	33 (75)	38 (86.4)	31 (70.5)	39 (88.6)	0.59 (0.34)	52.25 (19.12)
***P*** **-value**		0.119	0.305	0.768	0.068	0.068	**0.002** ^*^	**<0.001** ^*^
**Monthly family income**								
<15,000 taka	65 (15.3)	56 (86.2)	61 (93.8)	62 (95.4)	43 (66.2)	62 (95.4)	0.29 (0.43)	34.21 (15.72)
15,000–30,000 taka	200 (47.2)	157 (78.5)	158 (79)	171 (85.8)	143 (71.5)	163 (81.5)	0.40 (0.46)	38.66 (16.68)
31,000–45,000 taka	113 (26.7)	86 (76.1)	87 (77)	97 (85.8)	82 (72.6)	90 (79.6)	0.43 (0.47)	45.38 (19.31)
>45,000 taka	46 (10.8)	37 (80.4)	39 (84.8)	39 (84.3)	32 (69.6)	90 (79.6)	0.38 (0.47)	44.76 (20.32)
***P*** **-value**		0.445	**0.027** ^*^	0.189	0.818	**0.021** ^*^	0.316	**0.001** ^*^
**Occupation**								
Employed	198 (46.7)	150 (75.8)	158 (79.8)	170 (85.9)	128 (64.6)	168 (84.8)	0.47 (0.42)	42.22 (17.69)
Unemployed	226 (53.3)	186 (82.3)	182 (82.7)	199 (88.1)	172 (76.1)	188 (83.2)	0.31 (0.48)	38.86 (18.31)
***P*** **-value**		0.097	0.437	0.502	**0.010** ^*^	0.642	**<0.001** ^**^	0.056
**Co-morbidity**								
No	58 (13.7)	43 (74.1)	44 (75.9)	50 (86.2)	31 (53.4)	47 (81)	0.54 (0.39)	45.32 (18.81)
Yes	366 (86.3)	293 (80.1)	301 (82.2)	319 (87.2)	269 (73.5)	309 (84.4)	0.37 (0.47)	39.65 (17.87)
***P*** **-value**		0.302	0.246	0.841	**0.002** ^*^	0.513	**0.004** ^*^	**0.035** ^*^
**Smoking**								
No	228 (53.8)	185 (81.1)	191 (83.8)	204 (89.5)	160 (70.2)	191 (83.8)	0.40 (0.43)	41.99 (18.99)
Yes	196 (46.2)	151 (77)	154 (78.6)	165 (84.2)	140 (71.4)	165 (84.2)	0.38 (0.48)	38.61 (16.84)
***P*** **-value**		0.299	0.17	0.106	0.777	0.908	0.574	0.055
**Tobacco habit**								
No	217 (51.2)	157 (72.4)	167 (77)	183 (84.3)	144 (66.4)	173 (79.7)	0.47 (0.44)	41.02 (17.13)
Yes	207 (48.8)	179 (86.5)	178 (86)	186 (89.9)	156 (75.4)	183 (88.4)	0.31 (0.47)	39.81 (19.05)
***P*** **-value**		**>0.001** ^**^	**0.017** ^*^	0.091	**0.042** ^*^	**0.015** ^*^	**<0.001** ^**^	0.096
**Receiving rehabilitation care**								
No	158 (37.3)	155 (98.1)	156 (98.7)	157 (99.4)	133 (84.2)	155 (98.1)	0.13 (0.35)	48.12 (10.30)
Yes	266 (62.7)	181 (68)	189 (71.1)	212 (79.7)	167 (62.8)	201 (75.6)	0.54 (0.45)	27.48 (10.30)
***P*** **-value**		**>0.001** ^**^	>0.001^**^	**>0.001** ^**^	**>0.001** ^**^	**>0.001** ^**^	**<0.001** ^**^	**0.001** ^*^
**Type of stroke**								
Ischemic	357 (84.2)	279 (78.2)	279 (78.2)	311 (87.1)	245 (68.6)	26 (82.9)	0.42 (0.45)	41.33 (18.38)
Hemorrhagic	67 (15.8)	57 (85.1)	57 (85.1)	58 (86.6)	55 (82.1)	60 (89.6)	0.21 (0.49)	35.64 (15.69)
***P*** **-value**		0.2	0.396	0.903	**0.026** ^*^	0.174	**0.002** ^*^	**0.009** ^*^
**Duration of stroke**								
1–3 months	280 (66)	227 (81.1)	227 (81.1)	249 (88.9)	186 (65.7	239 (85.4)	0.37 (0.46)	38.85 (17.04)
4–6 months	58 (13.7)	47 (81)	47 (81)	52 (89.7)	47 (81)	48 (82.2)	0.43 (0.41)	44.15 (18.77)
>6 months	86 (20.3)	62 (72.1)	28 (72.1)	68 (79.1)	69 (80.2)	69 (83.8)	0.43 (0.50)	43.05 (20.32)
***P*** **-value**		0.187	0.443	**0.048** ^*^	**0.006** ^*^	0.508	0.493	**0.040** ^*^
**Side the of the weakness of the body**								
Right side	191 (45)	154 (80.6)	154 (80.6)	166 (86.9)	129 (67.5)	160 (83.8)	0.39 (0.45)	41.41 (19.07)
Left side	223 (52.6)	172 (77.1)	172 (77.1)	193 (86.5)	161 (72.2)	186 (83.4)	0.40 (0.47)	40.21 (17.12)
Both side	10 (2.4)	10 (100)	10 (100)	10 (100)	10 (100)	10 (100)	0.12 (0.26)	26.30 (14.75)
***P*** **-value**		0.178	0.287	0.463	0.064	0.174	**0.019** ^*^	**0.035** ^*^
**Number of stroke episodes**								
1st	335 (79)	251 (74.9)	257 (76.7)	281 (83.9)	223 (66.6)	267 (79.7)	0.46 (0.44)	42.22 (18.17)
Recurrent	89 (21)	85 (95.5)	88 (98.9)	88 (98.9)	77 (86.5)	89 (100)	0.14 (0.43)	33.69 (16.15)
***P*** **-value**		**<0.001** ^**^	<0.001^**^	**<0.001** ^**^	**<0.001** ^**^	<0.001^**^	**<0.001** ^**^	**0.001** ^*^
**Cohabitant situation**								
Spouse and children	321 (75.7)	247 (76.9)	253 (78.8)	274 (85.4)	219 (68.2)	264 (83.2)	0.44 (0.44)	41.55 (18.69)
Others	103 (24.3)	89 (86.4)	92 (89.3)	95 (92.2)	81 (78.6)	92 (89.3)	0.23 (0.49)	36.96 (15.62)
***P*** **-value**		**0.039** ^*^	**0.017** ^*^	0.071	**0.043** ^*^	0.089	**<0.001** ^**^	**0.015** ^*^
**Care provider**								
Spouse and children		170 (73)	176 (75.5)	191 (82)	153 (65.7)	189 (81.1)	0.49 (0.42)	42.70 (19.23)
Others	233 (55)	166 (86.9)	169 (88.5)	178 (93.2)	147 (77.0)	167 (87.4)	0.26 (0.47)	37.67 (16.20)
***P*** **-value**	191 (45)	**0.001** ^*^	**0.001** ^*^	**0.001** ^*^	**0.011** ^*^	0.078	**<0.001** ^**^	**0.014** ^*^
**Medical expenses are provided by**								
Self/Spouse	139 (32.8)	100 (71.9)	105 (75.5)	117 (84.2)	79 (56.8)	115 (82.7)	0.53 (0.43)	46.38 (19.14)
Children	254 (59.9)	207 (81.5)	209 (82.3)	221 (87)	195 (76.8)	210 (82.7)	0.33 (0.46)	38.14 (17.06)
Others	31 (7.3)	29 (93.5)	31 (100)	31 (100)	26 (83.9)	31 (100)	0.22 (0.46)	32.45 (14.04)
***P*** **-value**		**0.011** ^*^	**0.006** ^*^	0.06	**>0.001**	**0.042** ^*^	**<0.001** ^**^	**<0.001** ^*^
**Assistive device use**								
No	316 (74.5)	237 (75)	244 (77.2)	266 (84.2)	201 (63.6)	254 (80.4)	0.49 (0.41)	42.14 (18.60)
Yes	108 (25.5)	99 (91.7)	101 (93.5)	103 (95.4)	99 (91.7)	102 (94.4)	0.09 (0.47)	35.40 (15.48)
***P*** **-value**		**<0.001** ^**^	**<0.001** ^**^	**0.003** ^*^	**<0.001** ^**^	**0.001** ^**^	**<0.001** ^**^	**<0.001** ^*^

EQ-5D, European Quality of Life Scale-5 Dimensions; VAS, visual analog scale; SD; standard deviation. The bold values indicates the significant values. ^*^p <0.05, ^**^p <0.001.

### 3.4 EQ-5D-5L utility index and VAS Scores

The respondents' age, marital status, education, occupation, co-morbidity, and tobacco intake, stroke type, number of stroke episodes, receiving rehabilitation, cohabitation situation, care provider, medical expanse providers, and use of assistive devices had significant associations with the EQ-summary index (all *P*-values <0.05). However, there was no significant association between the EQ-utility index and respondents' residence, monthly family income, smoking habit, or duration of stroke. The details are also presented in Table 3.

### 3.5 Predictors that influence each dimension of EQ-5D among stroke survivors

The results from the multiple binary logistic regression analysis revealed that stroke survivors who were widowed or single faced higher difficulties in the EQ-mobility dimension (AOR = 1.24, 95% CI = 0.35–4.45; *p* = 0.74) and pain/discomfort dimension (AOR = 2.85, 95% CI = 0.85–9.27; *p* = 0.083) compared to married survivors.

Those without rehabilitation services experienced significantly greater difficulty across various dimensions, with odds ratios (AOR) as follows: (29.37, 95% CI = 8.85–97.50; *p* = <0.001) for mobility, (43.21, 95% CI = 10.02–186.41; *p* = 0.001) for self-care, (43.47, 95% CI = 5.88–321.65; *p* = 0.001) for usual activities, and (4.35, 95% CI = 2.45–7.71; *p* = 0.001) for pain/discomfort.

Individuals with co-morbidity faced significantly greater challenges in the pain/discomfort dimension (AOR = 2.64, 95% CI = 1.32–5.28; *p* = 0.006) than those without co-morbidity. Stroke survivors with recurrent strokes had significantly higher difficulty in the mobility (AOR = 4.94, 95% CI = 1.63–14.98; *p* = 0.005), self-care (AOR = 19.15, 95% CI = 2.51–146.57; *p* = 0.004), and usual activities (AOR = 15.66, 95%CI = 1.97–124.37; *p* = 0.009) dimensions compared to those with a single stroke episode. See Table 4 for details.

**Table 4:** Multivariable logistic regression: factors that influence each dimension of EQ-5D among stroke survivors.

	**Mobility**	**Self-care**	**Usual activities**	**Pain/discomfort**	**Anxiety/ Depression**
	**AOR (95% Cl)**	**AOR (95% Cl)**	**AOR (95% Cl)**	**AOR (95% Cl)**	**AOR (95% Cl)**
**Age(years)**					
20–40	Ref	–	–		–
41–60	(–) 0.35 (−0.11 to 0.93)^*^				
61-80	(–) 0.44 (−0.137 to 1.37)				
**Marital status**					
Married	Ref	–	–	Ref	
Unmarried/widow	1.24 (0.35–4.45)			2.85 (0.88–9.27)	
**Tobacco habit**					
No	Ref			Ref	
Yes	2.00 (1.11–3.62)^*^	1.25 (0.68–2.31)		1.40 (0.84–2.33)	
**Type of stroke**					
Ischemic	–	–	–	Ref	–
Hemorrhagic				1.83 (0.83–4.04)	
**Duration of stroke**					
1–3 months	–	–	Ref	Ref	–
4–6 months			(–) 0.93 (0.34–2.53) ^*^	2.87 (1.27–6.51)^*^	
>6 months			(–) 0.33 (0.15–0.72)^*^	3.082 (1.50–6.36)^*^	
**Number of stroke episodes**					
Recurrent	Ref	Ref	Ref	–	–
1st	4.94 (1.63–14.98)^*^	19.15 (2.51–146.57)^*^	15.66 (1.97–124.37)^*^		
**Co-morbidity**					
No	–	–	–	Ref	–
Yes				2.64 (1.32–5.28)^*^	
**Receiving of rehabilitation**					
Yes	Ref	Ref	Ref	Ref	Ref
No	29.37 (8.85–97.50)^**^	43.21 (10.02–186.41)^**^	43.47 (5.88–321.65)^**^	4.35 (2.45–7.71)^**^	20.28 (5.96–69.05)^**^
**Cohabitant situation**					
Spouse and children	Ref	Ref	**–**	**–**	**–**
Others	0.75 (0.22–2.52)	1.21 (0.47–3.32)	**–**	**–**	**–**
**Care providers**					
Spouse and children	Ref	Ref	Ref	Ref	–
Others	2.20 (0.99–4.91)^*^	2.11 (0.93–4.81)	2.93 (1.44–5.96)^*^	1.29 (0.67–2.49)	
**Medical expenses provider**					
Self/spouse	–	–	–	Ref	–
Children only	–	–	–	1.85 (1.04–3.61)^*^	
Others	–	–	–	4.53 (1.39–15.11)^*^	
**Assistive device use**					
No	Ref	Ref	Ref	Ref	Ref
Yes	3.66 (1.63–8.16)^*^	3.55 (1.45–8.73)^*^	2.92 (1.1.05–8.11)^*^	5.52 (2.48–12.28)^**^	3.72 (1.43-9.65)^*^

EQ-5D, European Quality of Life Scale-5 Dimensions; AOR, Adjusted Odd Ratio; CI, Confidence Interval; SD, standard deviation.

^*^Statistically significant at <0.05; ^**^Statistically significant at <0.001.

## 4 Discussion

This study aimed to evaluate factors influencing the HRQoL in stroke survivors who underwent rehabilitation at four specific tertiary-level hospitals in Bangladesh. The study found significant association between mobility and participants' home location, smoking habits, and co-morbidities. Similarly, the self-care dimension showed notable associations with residential location, smoking habits, co-morbid conditions (like diabetes), use of rehabilitation services, and stroke duration. Additionally, the pain/discomfort component was significantly linked to age, co-morbidities, medical expenditure, and use of assistive equipment. The study revealed noteworthy challenges among participants, with the most prevalent issues being difficulties in engaging in regular activities, followed by anxiety or depression, challenges in performing personal self-care, mobility issues, and the experience of bodily pain. Our study's results align with a previous Malaysian study, highlighting comparable outcomes across different domains of HRQoL (Wong et al., 2021). A study in Poland (Jarosławski et al., 2020) also revealed parallels, with a majority of patients facing difficulties in multiple HRQoL domains. Specifically, 57.3% reported mobility issues, 40.3% encountered challenges in regular activities, 63.2% experienced pain or discomfort, 59% reported anxiety or depression symptoms, and 32.2% faced difficulties in self-care.

The study revealed that the EQ summary index and EQ self-reporting VAS score were 0.39 and 40.43, respectively. These values were considerably lower than those reported in studies conducted in Malaysia (Mei et al., 2022), Singapore (Yeoh et al., 2018), the USA (Xie et al., 2006) and in Germany (Sadlonova et al., 2021). This difference may be attributed to Bangladesh's classification as a low- and middle-income country (LMIC), with per capita expenditures on medical care around US$ 11, significantly below the World Health Organization's recommended range of $30–$40 per individual (Hameed et al., 2021). Additionally, distinct crosswalk value sets, along with socio-demographic and cultural differences (Wang and Langhammer, 2018; Wang et al., 2019), may contribute to this variation. The EQ self-care component exhibited strong correlations with variables such as marital status, monthly family income, tobacco use, caregivers, and medical expense providers in our study. Lourenço et al. reported in their study that survivors who were divorced showed higher mean scores on physical dimensions compared to survivors who were married or widowed. Individuals who had received a greater number of years of education and came from families with higher income levels exhibited higher levels of perception in all aspects of their HRQoL. In contrast, retired individuals who have survived serious events and those who still require caregiver assistance reported a worse view of their overall quality of life in multiple domains (Lourenço et al., 2021).

The study reveals a significant association between co-morbidities and the mobility dimension, particularly with diabetes. Multiple co-morbidities were also associated with the pain/discomfort dimension, specifically hypertension and hypertension with diabetes. These findings align with previous findings in Malaysia, emphasizing a substantial correlation between mobility and various co-morbidities (Wong et al., 2021). Furthermore, a significant correlation is found between the frequency of stroke occurrences and the overall wellbeing and life satisfaction in stroke survivors. Similar results were reported in the same Malaysian study, indicating that recurrent stroke episodes are linked to reduced self-care activities and increased susceptibility to pain and suffering (Wong et al., 2021). In an investigation of the effect of comorbidities on the functional outcome of patients, Simić-Panić et al. (2018) found that individuals with more comorbidities had a poorer prognosis after a stroke.

This study emphasizes the widespread prevalence of depression and anxiety among participants, specifically examining their connection to stroke—a debilitating brain disorder with immediate and profound impacts on individuals' lives. The consequences extend to both physical and psychological wellbeing, significantly affecting overall health. A study among stroke survivors in Bangladesh reported that 37.7% participants had low psychological quality of life (Sarkar et al., 2023). In Jordan, a cross-sectional study showed that 74.5% of stroke survivors experienced depression, 52.9% experienced anxiety, and 68% experienced stress (Almhdawi et al., 2020). A separate investigation in Ethiopia found a prevalence rate of 49.44% for anxiety or depression (Zemed et al., 2021). A cross-sectional study conducted in Bangladesh with 164 post-stroke patients revealed that 70% of the patient's experienced depression, with 32% having severe depression. Living in a joint family, being unable to do everyday tasks on one's own, and having comorbid dysphasia and hypertension were variables substantially related to depression reported in the study (Ariful Islam et al., 2016).

Moreover, 62.2% of stroke survivors were below 60 years old. This study highlights that individuals who experienced strokes in rural areas exhibit a greater prevalence of anxiety and depressive symptoms compared to those in metropolitan areas. These disparities in outcomes may stem from lower education levels and limited access to healthcare services. In Bangladesh, the majority of stroke rehabilitation facilities are located in large cities and are supported by both public and private sources (Venketasubramanian and Mannan, 2021). Consequently, integrating cognitive behavior therapy, problem-solving therapy, and counseling into stroke rehabilitation programs is crucial (Lee et al., 2021).

This study indicate that a notable number of participants faced difficulties in performing daily tasks such as housework, family and leisure activities, as well as essential activities like self-care and mobility. 75.3% of stroke survivors had low physical quality of life reported earlier study in Bangladesh (Sarkar et al., 2023). The root cause of these difficulties may be attributed to the insufficient availability of rehabilitation facilities in Bangladesh, thereby leading to suboptimal health services (Al Imam et al., 2022). Improving activities of daily living and fostering functional independence have demonstrated a positive influence on the overall quality of life for individuals who have experienced a stroke (Kim et al., 2014). Additionally, it is important to highlight that the COVID-19 epidemic may adversely affect the EQ-5D-5L dimension in individuals who have experienced a stroke.

The study utilized the Modified Rankin Scale (mRS) to assess participants disability. Additionally, comparing mRS with VAS self-reporting scores indicated that stroke survivors with lower disability levels reported higher VAS scores. A prior study in Pakistan reported similar findings (Khalid et al., 2016). A population-based study in Northeast China reported disability rate at 3-year follow-up of 46.7%. They found age, neurological deficits, cognitive function, depression, and social support as associated factor (Lv et al., 2021).

This study has brought to light a significant revelation regarding individuals who have experienced a stroke and subsequently undergone rehabilitation services. Notably, these individuals demonstrate significantly elevated levels of EQ-5D ranking. Antecedent research endeavors have further elucidated that participation in post-stroke rehabilitation represents a promising intervention. This intervention holds the potential to enhance the overall higher-order HRQOL among stroke survivors. These collective findings emphasize the profound impact of rehabilitation services on the subjective wellbeing and health outcomes of individuals who have experienced a stroke, thereby emphasizing the imperative role of such interventions in the holistic recovery process (Edwards and Connell, 2003; Chen et al., 2011; Rancic et al., 2020; Bisevac et al., 2022).

### 4.1 Clinical Implications and significance in public health

The study highlights key factors influencing HRQoL in post-stroke patients in Bangladesh. The study underscores the importance of tailored interventions, emphasizing family support, tobacco cessation, recurrent stroke prevention, and effective rehabilitation services. The insights offer healthcare professionals a basis for developing targeted post-stroke care strategies, potentially enhancing patient outcomes and HRQoL. Public health initiatives should address these factors to reduce stroke-related disability, improve HRQoL, and contribute to the overall wellbeing of the population. Addressing broader determinants of health, such as socioeconomic status and access to healthcare, is essential. Implementing these policies can significantly enhance stroke prevention, management, and rehabilitation in Bangladesh, aligning with the study's findings and underscoring its practical significance. From a policy perspective, the findings suggest practical implications such as integrating the promotion of family involvement in rehabilitation with appropriate training and resources into national health policies. Prioritizing tobacco cessation programs to reduce recurrent strokes, emphasizing routine screening and management of risk factors like hypertension and diabetes, and expanding access to comprehensive rehabilitation services are also crucial.

### 4.2 Strength and limitations

In this pioneering study, we present the first exploration of the health-related quality of life (HRQOL) among stroke survivors in Bangladesh. Employing a questionnaire administered through interviews, this research sought to enhance data precision. However, it is crucial to acknowledge several limitations inherent in the current investigation. Firstly, the study adopts a cross-sectional design and relies on non-random convenience sampling methods for participant selection. The sample exclusively consists of stroke survivors receiving treatment within the physiotherapy and rehabilitation departments of four specific tertiary-level institutions in Bangladesh. This renders generalizability to all stroke survivors in Bangladesh challenging. Convenience sampling may introduce biases by favoring easily accessible or willing participants, potentially excluding certain demographics and impacting overall representativeness. While the questionnaire has been previously validated and widely used, further study-specific validation could enhance the reliability and applicability of the findings in similar future research. Furthermore, the concurrent occurrence of a coronavirus epidemic during the investigation introduced substantial disruptions to health and rehabilitation services. While acknowledging the potential influence of these disruptions on our study outcomes, we regrettably lacked the means to comprehensively assess their impact.

Considering these constraints, it is imperative to interpret the findings cautiously. To advance our understanding of the factors influencing HRQOL among stroke survivors, future research endeavors should adopt a longitudinal approach. The HRQoL of stroke patients can be better understood through a cohort study with a minimum 2-year follow-up. Follow-up studies will enhance our understanding of HRQoL in post-stroke patients in Bangladesh by focusing on physical, emotional, and social impacts, rehabilitation program effectiveness, socioeconomic influences, and healthcare policy efficacy. Addressing these areas will provide comprehensive insights and inform targeted interventions to improve patients' lives. This would facilitate a comprehensive exploration of the parameters intricately linked to HRQOL in individuals who have experienced a stroke, thereby contributing to a more nuanced and generalizable body of knowledge in this field.

## 5 Conclusion

In conclusion, our study sheds light on the critical factors influencing HRQoL in post-stroke patients within the unique context of Bangladesh. The identified key determinants—family support, tobacco cessation, prevention of recurrent stroke, and effective rehabilitation services—underscore the need for targeted interventions to enhance the HRQoL of individuals recovering from stroke in this region. This study not only adds valuable insights into the intricate interplay of factors influencing HRQoL in post-stroke patients but also provides actionable recommendations for clinicians, researchers, and policymakers. Embracing a comprehensive, multidimensional approach is essential to improve the lives of stroke survivors in Bangladesh and, potentially, in similar contexts globally. Furthermore, the call for longitudinal studies to corroborate our findings underscores the importance of ongoing research to refine our understanding of post-stroke dynamics in this population. Longitudinal data will provide a more nuanced perspective on the evolution of HRQoL over time and enable the development of more effective and targeted interventions.

## Data availability statement

The original contributions presented in the study are included in the article/supplementary material, further inquiries can be directed to the corresponding author.

## Ethics statement

This study obtained ethical clearance from the Ethics Committee of the Bangladesh Open University (BOU/SST/MDMR/146/17/376). The studies were conducted in accordance with the local legislation and institutional requirements. The participants provided their written informed consent to participate in this study.

## Author contributions

MI: Conceptualization, Data curation, Formal analysis, Investigation, Methodology, Project administration, Resources, Validation, Visualization, Writing – original draft, Writing – review & editing. SA: Conceptualization, Data curation, Formal analysis, Investigation, Methodology, Project administration, Resources, Software, Validation, Visualization, Writing – original draft, Writing – review & editing. SK: Writing – original draft, Writing – review & editing. MR: Writing – original draft, Writing – review & editing. SN: Writing – original draft, Writing – review & editing. SC: Writing – original draft, Writing – review & editing. MU: Conceptualization, Data curation, Formal analysis, Methodology, Software, Validation, Visualization, Writing – original draft, Writing – review & editing. MK: Conceptualization, Data curation, Formal analysis, Methodology, Resources, Software, Supervision, Validation, Visualization, Writing – original draft, Writing – review & editing.
